# Quality of life improving after propranolol treatment in patients with Infantile Hemangiomas

**DOI:** 10.1186/s13052-022-01334-2

**Published:** 2022-08-04

**Authors:** Marco Pensabene, Maria Rita Di Pace, Fabio Baldanza, Francesco Grasso, Maria Patti, Maria Sergio, Simona La Placa, Mario Giuffre’, Gregorio Serra, Alessandra Casuccio, Marcello Cimador

**Affiliations:** Department of Health Promotion, Mother and Child Care, Internal Medicine and Medical Specialties, University of Palermo, Palermo, Italy

**Keywords:** IH, Quality of life, Pediatric benign tumors, Propranolol, Questionnaire

## Abstract

Infantile hemangiomas may affect the quality of life (QoL) of patients and their family members, as anxiety and worry may commonly occur in parents, also linked to the social adversion they experience. We underline the beneficial impact of oral propranolol therapy on QoL of patients with infantile hemangiomas (IH) and of their relatives. A specific questionnaire measuring QoL was administered to parents of IH patients at beginning and end of a treatment with oral propranolol. Different aspects were investigated: site of the lesion, age of patients at starting therapy, length of treatment, occurrence of adverse effects and persistence/recurrence of the vascular anomaly. In all cases the questionnaire revealed a significant improvement of QoL, which was independent from all analyzed factors. It showed that oral propranolol administration in these patients combines optimal clinical results with relevant improvement of QoL, especially in cases of early management. The improvement of QoL seems unrelated to site of lesion, timing and duration of therapy, occurrence of drug-related adverse effects and persistence/recurrence of disease.

## Background

Infantile Hemangiomas (IH) are quite common benign cutaneous tumors, occurring in 5–10% of infants [1]. IHs are not usually evident at birth, but a proliferative phase commonly begins during the first month of life. They show a complete growth in 80% of patients at 5 months of age. However, they may enlarge until 9–12 months and, in rare cases, a continuous growth is observed up to 24 months [1]. This phase is followed by a slow and gradual involution, which is usually completed within 10 years of life, and mostly within age 4 [1]. Despite the benign nature of IH, some complications may occur during their natural history: ulceration, bleeding, visual impairment or airways obstruction if periorbital region or upper airways are involved respectively. They are strongly related to both size and localization of the vascular anomaly [2]. Scars and disfigurement are also common. The introduction of oral propranolol therapy has radically changed the clinical course of IH, allowing an early interruption of the evolutive phase and, moreover, accelerating the involution of the lesion [3].

Clinicians must consider, even in uncomplicated cases, the risk of permanent disfigurement and the following potential psycho-social impact on patients and their families [4, 5]. Therefore, a validated instrument for QoL evaluation in subjects affected by IH has previously been developed, according to the clinical characteristics of hemangiomas which mainly have a psychological impact on parents [2]. We aimed to evaluate the improvement of QoL in parents of a series of children with IH treated with oral propranolol.

## Patients and methods

A total number of 99 patients with IH have been observed in our Department from January 2016 to December 2019. Of these subjects, 94 had high risk IH, eligible to oral propranolol therapy according to AAP guidelines [6], while 5 presented with known or suspected cardiovascular or cerebrovascular conditions not suitable for propranolol therapy, and were then excluded from our study. The therapeutic course to which our patients underwent ended after 12 months therapy, or 10 months if complete clinical regression was observed before. A validated questionnaire for QoL evaluation was administered to the parents of such patients. However, those with incomplete therapeutic course and/or uncertain parental compliance to therapy (*n* = 39), and with an ongoing second course due to relapsing or persistent IH (*n* = 28) were excluded, and the questionnaire based analysis finally concerned and was conducted on remaining 26 patients’ parents.

Oral propranolol was administered at the therapeutic dose of 3 mg/kg/day divided twice in a day. The therapeutic range was reached with weekly increase, starting from 1 mg/kg/day. In order to evaluate patient tolerance to the dose increase, monitoring of blood pressure, heart rate, glycaemia and blood oxygen level analysis were regularly investigated before and during treatment. Once the therapeutic dose was reached (3 mg/Kg/day), patients were clinically evaluated monthly. According to IH localization, three main body districts were identified, defining the following groups: 1) face/neck; 2) acral regions (limbs, hands, genital and perineal ones); 3) back/abdomen/thorax.

The specific IH QoL questionnaire was administered to enrolled families in two different moments: at the beginning and at the end of the first course of therapy. IH-QoL was established by a total score ranging from 0, meaning no impact on QoL, to 116 considered as severe effect, which resulted from different sub-scores. These latter, measured four different aspects of QoL related both to child and parents: child physical symptoms – CPS (score range 0–16) and social interactions – CSI (score range 0–20); and parent psychosocial functioning—PPF (score range 0–40) and emotional functioning—PEF (score range 0–40). The questionnaire is reported in Table 1.

**Table 1:** Questionnaire used in the present study (adapted from Chamlin SL et al. [2])

1. My child has pain because of this hemangioma. (CPS)
2. My child seems sickly or prone to illness because of the hemangioma. (CPS)
3. My child has trouble sleeping because of the hemangioma. (CPS)
4. Because of the hemangioma my child has problems being soothed or comforted when crying. (CPS)
5. Children seem to avoid touching or playing with my child because of his/her hemangioma. (CSI)
6. My child’s hemangioma makes me feel sad or depressed. (PEF)
7. I am disappointed that my child has this hemangioma. (PEF)
8. I experience more headaches than usual as a result of my child’s hemangioma. (PSF)
9. My child’s hemangioma makes me feel anxious or nervous. (PEF)
10. I am bothered when strangers stare at my child. (CSI)
11. I am embarrassed by the way my child looks because of his/her hemangioma. (PEF)
12. I am worried that in the future my child will not make friends as easily because of the hemangioma. (PEF)
13. I blame myself or my child’s other parent that my child has this hemangioma. (PSF)
14. The hemangioma has affected how confident I feel about my child’s medical care. (PEF)
15. I get worried when I see changes in my child’s hemangioma. (PEF)
16. I have been frustrated with my child’s medical care for the hemangioma. (PEF)
17. I am bothered that my child needs to be watched more closely at home because of the hemangioma. (PSF)
18. I feel physically weak as a result of my child’s hemangioma. (PSF)
19. I am bothered when strangers offer opinions or ask questions about my child’s hemangioma. (CSI)
20. My child’s hemangioma affects our social life. (PSF)
21. My child’s hemangioma has strained my relationship with my spouse or partner. (PSF)
22. I have been accused of child abuse because of my child’s hemangioma. (CSI)
23. Our family is less likely to go to public places (e.g., grocery store) because of the hemangioma. (PSF)
24. My child’s hemangioma affects my or my spouse/partner’s work due to missed time. (PSF)
25. I am bothered that children touch or comment on my child’s hemangioma. (CSI)
26. I worry about my child based on information I read on the Internet. (PEF)
27. I worry about side effects of the medication(s) used to treat my child’s hemangioma. (PEF)
28. I feel too tired to do the things I like to do because of my child’s hemangioma. (PSF)
29. I have felt sick to my stomach as a result of my child’s hemangioma. (PSF)

*Abbreviations: CPS *child physical symptoms, *CSI *child social interactions, *IH-QoL *Infantile Hemangioma Quality-of-Life, *PEF *parent emotional functioning, *PSF *parent psychosocial functioning

In all subscores, higher indexes indicated lower QoL related with the corresponding aspect analyzed. Differences in QoL measuring scores, before and after oral propranolol treatment, were compared in terms of global scoring and sub-scoring. The following parameters, with a potential impact on QoL during therapy, were also investigated: 1) area of lesions; 2) age of patient; 3) treatment duration; 4) occurrence of adverse effects; 5) recurrence/persistence of disease.

Statistical analysis of quantitative and qualitative data, including descriptive statistics, was performed for all items. Continuous data were expressed as mean ± standard deviation (SD), unless otherwise specified. The independent Student's t-test and Univariate analysis of variance (ANOVA) test were used to compare parametric variables between the different groups. The paired samples Student's t-test was used to compare the pre-post questionnaire scores. Data were analyzed by IBM SPSS Software 22 version (IBM Corp., Armonk, NY, USA). All *p*-values were two-sided, and *p* < 0.05 was considered statistically significant.

## Results

After the exclusion of subjects with potentially misleading conditions, we collected and analyzed data of a sample of 26 patients. Mean age at starting therapy was 2.27 months (range 1–6), while mean duration was 10.50 months (range 6–15 months). All patients reached the therapeutic dose of 3 mg/kg/day within the first month of treatment.

No major adverse effects were reported. Three patients (mean age 4.2 months) experienced minor adverse effects after propranolol administration, namely sleep disturbances during the first month of therapy. In these patients the daily dose was divided into three administrations, minimizing thus the occurrence of insomnia and restlessness.

No complications were reported during the study period, although 6 patients presented with recurrence/persistence of disease, and were then scheduled for a second treatment course.

Regarding to localizations, 18 patients showed IH of the face/neck; 5 patients had acral IH, while 3 patients had thorax/abdomen/back involvement.

QoL was measured with the specific questionnaire. All families reported a reduction of the QoL scores, meaning a lower impact of IH on familiar QoL. Actually, the mean total pre-therapy score was 29.38/116 (range 6–47/116), and the score decreased to 5.68/116 at the end of treatment (range 0–22/116) with a statistically significant difference (*p* < 0.0005). All subscores became as well significantly lower at the end of therapy (Table 2).

**Table 2:** Results of IH QoL questionnaire are summarized. Global score and sub-scores significantly improved after administration of oral propranolol

	**Pre-treatment (mean)**	**Post-treatment (mean)**	***P*-value**
**Global result (0–116)**	29.38	6.58	< 0.0005
**Child Physical Symptoms (0–16)**	1.27	0.04	0.002
**Child social interaction (0–20)**	4.77	1.46	0.0005
**Parental psychosocial functioning (0–40)**	3.15	0.27	0.0005
**Parental emotional functioning (0–40)**	20.19	4.81	0.0005

The Child-physical-symptoms sub-score changed from a mean of 1.27/16 (range 0–7/16) to 0.04/16 (range 0–1/16); the child-social-interaction sub-score decreased from 4.77/20 (range 0–12/20) to 1.46/20 (range 0–9/20); the parental-psychosocial-functioning sub-score from 3.15/40 (range 0–9/40) to 0.27/40 (range 0–2/40); the parental emotional functioning sub-score from 20.19/40 (range 6–31/40) to 4.81/40 (range 0–15/40). All reported differences were found to be statistically significant. Comparing the improvement of QoL, no significant differences relating to area involved by the lesion, age of patients (2.27 month, range 1–6), and treatment duration (10.5 months, range 6–15, SD 2.52) were found. Furthermore, recurrence/persistence of the vascular anomaly, in addition to therapy-related adverse effects, did not negatively affect QoL (Tables 3, 4 and 5).

**Table 3:** Changes in pre- and after therapy scores, according to the district involved. No significant differences were observed

District	N patients	Δ-Global result	Δ-Child Physical Symptoms	Δ-Child social interaction	Δ-Parental psychosocial functioning	Δ-Parental emotional functioning
1	18	22.0	1.28	3.28	2.55	14.88
2	5	28.8	1.0	4.6	4.40	18.80
3	3	17.67	1.33	1.33	2.33	12.66
Total	26	22.81	1.23	3.30	2.88	15.38
*p*-value (between groups)	/	0.331	0.995	0.463	0.341	0.389

**Table 4:** Recurrence/persistence of the lesions does not decrease the improvement of QoL after oral propranolol treatment

	**No persistence/ recurrence**	**Persistence/ recurrence**	***P*-value**
Δ-Global result	22.05	27.0	0.412
Δ-Child Physical Symptoms	1.09	2.0	0.374
Δ-Child social interaction	3.45	2.5	0.628
Δ-Parental psychosocial functioning	2.54	4.75	0.111
Δ-Parental emotional Functioning	14.95	17.75	0.444

**Table 5:** Adverse effects do not decrease the improvement of QoL after oral propranolol treatment

	**Adverse effects**	**No Adverse effects**	***P*-value**
Δ-Global result	23.0	21.33	0.808
Δ-Child Physical Symptoms	1.13	2.0	0.453
Δ-Child social interaction	3.39	2.66	0.745
Δ-Parental psychosocial functioning	3.08	1.33	0.268
Δ-Parental emotional Functioning	15.39	15.33	0.989

## Discussion and conclusions

IH may have relevant impact on the QoL of patients and their parents, and social and psychological implications have been reported [2, 5, 6]. Lack of prenatal diagnosis and progressive enlargement of the lesion during the first few months of life may cause anxiety, worry, and social rejection. These reactions may be amplified when IH involves the head/neck, in case of multiple lesions, or if complications occur, as ulceration or bleeding leading to scarring.

A specific instrument for QoL evaluation of patients with IH and of their parents has been described and validated [2]. This is a questionnaire divided into 4 different areas, involving different aspects of life: patient physical symptoms and social interaction, parental social and emotional functioning. Each item could be studied separately, but all factors concur to the global score that summarizes the impact of IH on QoL (Table 1).

Oral propranolol administration recently gained increasing relevance in the therapeutic approach to IH, and currently it is widely used as first-line therapy in many Pediatric Centers, showing higher effectiveness than conventional treatment with steroids [7], in addition to lower rates of severe complications related to chronic administration [8, 9].

Our study confirms the effectiveness of this drug in the treatment of IH and, moreover, underlines the better QoL in families who choose to administer oral propranolol.

QoL evaluation score of all patients of our series showed a statistically significant decrease (global score *p* < 0.000), meaning a substantial improvement of QoL at the end of treatment.

The reduction of the different sub-scores was more marked in the *patient social interaction* (*p* < 0.000), *parental social functioning* (*p* < 0.000) and *parental emotional functioning* (*p* < 0.000) items. Although less relevant, also the aspects related to *child physical symptoms* showed a significant difference in the improvement of the sub-score (*p* = 0.002).

**Fig. 1:**
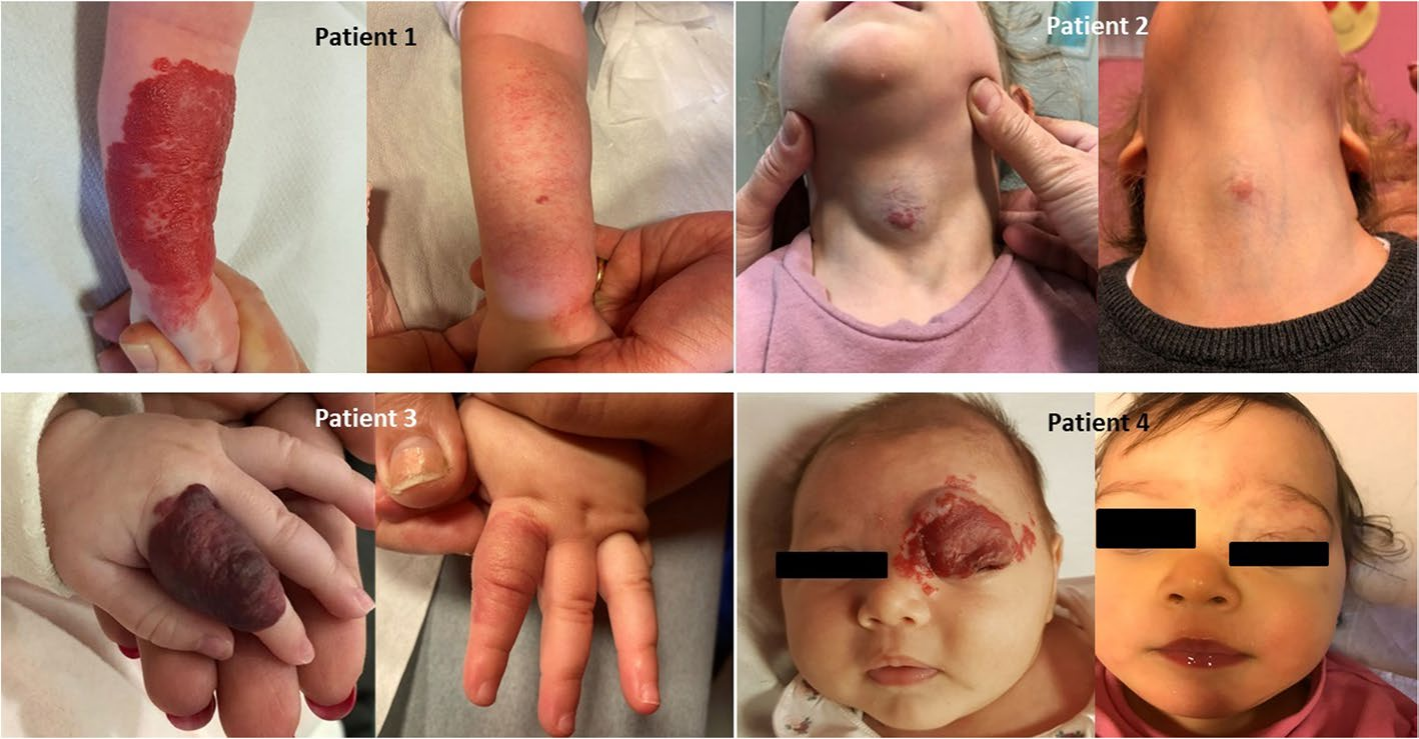
Evolution of IH involving different body districts in 4 of our patients after propranolol therapy

IH involving face or neck have shown higher negative impact on QoL in other previous studies [2], while in our series family QoL seems to improve regardless of the body district involved. Such “*missed*” difference may be due to the homogeneity of our study population, and to the early recruitment of our patients. In the present series (Fig. 1) most of patients started treatment in an early phase of the IH natural history, minimizing the aesthetic impact of the lesion and thus, the well-known psychological consequences for parents, documented as higher for IH of the face or neck.

Our observations confirm that an early management of patients with IH has a positive impact on the therapeutic effectiveness, and is able as well to prevent the worsening of IH-related QoL [2, 10]. The treatment of IH in reference centers seems to improve diagnostic and therapeutic results [11, 12], and may help parents to a better understanding of the disease, including both diagnostic course and therapy. Moreover, it allows parents to share personal experiences with other families living similar conditions [13].

The present study may have some limitations. First, the questionnaire is not validated in Italian [14]; secondly, the study population is small, leading to less strong conclusions and/or potential statistical bias. However, the strictly observed exclusion criteria conversely allowed to obtain a homogeneous population.

IH are quite common lesions. They significantly affect QoL of the families involved, and may also lead to potentially severe complications. Early management within reference centers allowing proper and prompt diagnosis, treatment and monitoring [15–18], should be guaranteed to affected subjects, in light of the known more favorable outcomes in addition to the positive effects on QoL.

## Data Availability

The datasets used and analyzed during the current study are available from the corresponding author on reasonable request.

## Abbreviations

IH: Infantile Hemangiomas
QoL: Quality of life
